# A General Local Reconstruction Approach Based on a Truncated Hilbert Transform

**DOI:** 10.1155/2007/63634

**Published:** 2007-06-18

**Authors:** Yangbo Ye, Hengyong Yu, Yuchuan Wei, Ge Wang

**Affiliations:** ^1^Department of Mathematics, University of Iowa, Iowa City, IA 52242, USA; ^2^CT Laboratory, Biomedical Imaging Division, VT-WFU School of Biomedical Engineering, Virginia Tech, Blacksburg, VA 24061, USA; ^3^CT Laboratory, Biomedical Imaging Division, VT-WFU School of Biomedical Engineering, Wake Forest University, Winston-Salem, NC 27157, USA

## Abstract

Exact image reconstruction from limited projection data has been a central topic in the computed tomography (CT) field. In this paper, we present a general region-of-interest/volume-of-interest (ROI/VOI) reconstruction approach using a truly truncated Hilbert transform on a line-segment inside a compactly supported object aided by partial knowledge on one or both neighboring intervals of that segment. Our approach and associated new data sufficient condition allows the most flexible ROI/VOI image reconstruction from the minimum account of data in both the fan-beam and cone-beam geometry. We also report primary numerical simulation results to demonstrate the correctness and merits of our finding. Our work has major theoretical potentials and innovative practical applications.

## 1. INTRODUCTION

Since its introduction in 1973 [1], X-ray CT has revolutionized radiography and become a cornerstone of all the modern hospitals and clinics. With development of sources, detectors, computers, and algorithms, X-ray CT is in a rapid transition from fan-beam to cone-beam geometry. On the daily basis, the state-of-the-art medical CT scanners routinely produce a huge amount of 2D, 3D, 4D, and even 5D (multiple energies) images of anatomy and functions with sub-mm spatial resolution, a few thousandth contrast resolution, and subsecond temporal resolution. On the other hand, the rapid development of small animal models, especially those with genetically engineered mice, has generated the critical needs for preclinical imaging. With refined CCD cameras and microfocus X-ray tubes, a number of micro-CT systems were constructed since the 1990s, reaching image resolutions between 10–100 *μ*m. Nevertheless, important and immediate biomedical studies still demand significantly better CT/micro-CT performance, so do industrial, homeland security, and other applications.

A public concern with X-ray CT is that the radiation dose is delivered to the patient during the CT scan. Annually, over 6 000 000 CT scans were performed in the US with 600 000 of those done on pediatric patients. The CT dose is the primary component in the radiation exposure to the US population. While CT studies account for only 4% of radiological procedures, they contribute nearly 40% of the average medical radiation dose. The contribution of CT to the average medical radiation dose level is expected to grow as the CT technology improves with multirow detectors and cone-beam designs. Therefore, there is a serious and increasing public concern over CT dose, particularly in the context of mass screening and pediatric imaging. The radiation dose to children from CT procedures is a particular concern since their risk of radiation-induced cancer is higher than that of adults, they have a longer lifetime for the cancer to be expressed and the effective dose they receive is typically larger than that received by adults for a comparable study [2, 3]. Because the radiation detriment is conservatively assumed to be linearly related to dose, there should be substantial health benefits on the overall US population from low-dose CT. As of this date, the dose reduction potential has not been systematically investigated in terms of algorithmic optimization, which we believe is an urgent issue we must address.

Similar negative arguments can be made for micro-CT studies of small animals, especially mice and rats. Currently, almost all of the human diseases have corresponding small animal models. Micro-CT has been widely used as a most valuable imaging tool in this regard. The nature of such small animal studies such as mouse studies requires higher spatial, contrast, and temporal resolution to be delivered periodically and even continuously. As a result, the increment in radiation dose becomes a major factor preventing more effective applications of micro-CT in this area. For example, to evaluate the heart and lungs, we need to depict the boarders of the lungs, lobes, sublobar segments, airways, vessels, as well as cardiac chambers, myocardium and dynamics. However, even the best micro-CT protocols and systems clearly fall behind our expectations, not only the involved radiation dose but also slow data acquisition.

Technically speaking, the limited data reconstruction strategy holds the promise to enhance the CT/micro-CT performance significantly. This strategy may reduce the X-ray radiation exposure and improve the data acquisition speed at the same time. The importance of performing exact image reconstruction from the minimum account of data has been recognized for long time. The first landmark achievement is the well-known fan-beam half-scan formula [4]. A relatively recent milestone is the fan-beam super-short-scan formula developed by Noo et al. [5]. Let *μ* $\left(\vec{r}\right)$ be a smooth function on a compact support Ω ⊂ ℝ^2^, with $\vec{r}$ = (*r*_1_, *r*_2_) and ℝ^2^ the 2D real space. Define the line integral

(1) $$p\left(s,\varphi \right)=\int_{ℛ}\mu \left(s\vec{u}\left(\varphi \right)+t{\vec{u}}^{⊥}\left(\varphi \right)\right)dt$$

for *s* ∈ ℝ and 0 < *Φ* < *π*, where $\vec{u}$ (*Φ*) = (cos *Φ*, sin *Φ*) and ${\vec{u}}^{⊥}$ (*Φ*) = (− sin *Φ*, cos *Φ*). *p*(*s*, *Φ*) can be extended to *Φ* ∈ ℝ by *p*(*s*,*Φ* + *π*) = *p*(−*s*, *Φ*). For a fixed *Φ*_0_, by Gel'fand and Graev [6] and Noo et al. [7], the backprojection data

(2) $$b\left(\vec{r_{0}}\right)=-\frac{1}{2\pi }\int_{\varphi_{0}}^{\varphi_{0}+\pi }\frac{\partial p\left(s,\varphi \right)}{\partial s}{|}_{s=\vec{r_{0}}\cdot \vec{u}\left(\varphi \right)}d\varphi$$

can be expressed as the Hilbert transform of *μ* along the line *L* through $\vec{r}$_0_ which is parallel to $\vec{n}$ = (−sin *Φ*_0_, cos *Φ*_0_:

(3) $$b\left(\vec{r_{0}}\right)=\frac{1}{\pi }\text{pv}\int_{R}\mu \left(\vec{r_{0}}-t\vec{n}\right)\frac{dt}{t}=\left(H_{L}\mu \right)\left(\vec{r_{0}}\right),$$

where “PV” represents the principal value. By the inversion formula of the finite Hilbert transform [8], the backprojection data can be inverted to reconstruct the function *μ*. In [7], Noo et al. proposed a sufficient condition for exact and stable ROI reconstruction from 2D limited data, which can be summarized as [9]:*“the function* *μ* *can be exact reconstructed at a point ${\vec{r}}_{0}$ if one can find a unit vector* $\vec{n}$ = (−sin *Φ*_0_ ,cos *Φ*_0_) *and a simply connected segmented* *L*_*μ*_ ⊂ *L* *of the line* *L* *parallel to* $\vec{n}$ *through* $\vec{r}$_0_ *such that* (i) *the segment* *L*_*μ*_ includes $\vec{r}$_0_ *and covers the whole support of* *μ* *along* *L*, that is, *μ* $\left(\vec{r}\right)$ = 0*for* $\vec{r}$ ∈ *L*\ *L*_*μ*_; *and* (ii) *for each* $\vec{r}$ ∈ *L*_*μ*_ *and each angle* *Φ* ∈ [*Φ*_0_, *Φ*_0_ + *π*] *the line integral* *p*(*s*, *Φ*) *are known for a neighborhood of* *s* = $\vec{r}$ · $\vec{u}$(*Φ*). ” In the cone-beam geometry, the groundbreaking work by Katsevich allows exact image reconstruction from truncated helical cone-beam data of less than two turns [10–12]. His results were further improved by a backprojection-filtration formulation in the helical cone-beam case [13] and its generalization [14–22], which permit transversely truncated data as well. Last year, Defrise et al. further strengthened their above-quoted sufficient condition by modifying (i) as “*the segment* *L*_*μ*_ *contains* $\vec{r}$_0_ *and at least one of its end points is outside the convex hull of the support of* *μ* *along* *L*” [9]. This latest finding represents the up-to-date record in the area of limited data reconstruction.

In this paper, we present a general ROI/VOI reconstruction approach using a truly truncated Hilbert transform on a line-segment inside a compactly supported object aided by partial knowledge on one or both neighboring intervals of that segment. As a result, the most flexible ROI/VOI reconstruction can be exactly performed in the fan-beam/cone-beam geometry. We are excited by numerous practical possibilities and associated benefits in image quality improvement and radiation dose reduction [23]. In Section 2, we will study the inverse problem of the truncated Hilbert transform and establish the uniqueness and stability of the solution. In Section 3, we will formulate a new sufficient condition for exact reconstruction of an ROI from limited data and propose a generalized reconstruction approach. In Section 4, we will implement our method and present representative simulation results. Finally, in Section 5, we will discuss the relevant issues and conclude the paper.

## 2. TRUNCATED HILBERT TRANSFORM WITH PARTIAL NEIGHBORING INFORMATION

In reference to [9], let us denote the 2D *μ* $\left(\vec{r}\right)$on certain line *L* as *f*(*x*), where *x* is the one-dimensional (1D) coordinate along the line *L*. Without loss of generality, we further assume that the support of *f*(*x*) on *L* is [−1,1]. Denote by

(4) $$g\left(x\right)=\left(Hf\right)\left(x\right)=\frac{1}{\pi }\text{pv}\int_{-1}^{1}f\left(y\right)\frac{dy}{x-y}$$

the Hilbert transform of *f*(*x*). By Tricomi [8], *f*(*x*) can be recovered from its Hilbert transform *g*(*x*) by

(5) $$\sqrt{1-x^{2}}f\left(x\right)=C_{f}+\frac{1}{\pi }\text{pv}\int_{-1}^{1}g\left(y\right)\sqrt{1-y^{2}}\frac{dy}{y-x},$$

where

(6) $$C_{f}=\frac{1}{\pi }\int_{-1}^{1}f\left(x\right)dx$$

is a known quantity. Our main contribution can be summarized in the following theorem.

**Theorem 1.**

**Figure 1:**
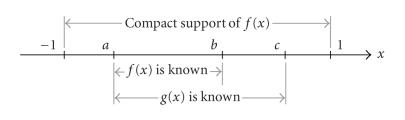
Setting for Theorem 1, where f (x) is supported on [−1, 1] and known on (*a*, *b*), while its Hilbert transform known on (*a*, *c*).

*a, b, c are three real numbers with −1 < a < b < c <1 (see Figure 1). A function* *f*(*x*) *supported on* [−1,1] *can be exactly reconstructed on* [*b*, *c*) *if* (i) *f*(*x*) *is known on* (*a*, *b*); (ii) *g*(*x*) *is known on* (*a*,*c*), *and* (iii) *the constant* *C*_*f*_ *is known (see Figure 1).*

**Proof.**

By (5), we have the inversion formula

(7) $$\begin{array}{c} \sqrt{1-x^{2}}f\left(x\right)=C_{f}+\frac{1}{\pi }\text{pv}\int_{-1}^{a}g\left(y\right)\sqrt{1-y^{2}}\frac{dy}{y-x}\\ \;\;\;\;\;\;\;\;\;\;+\frac{1}{\pi }\text{pv}\int_{a}^{b}g\left(y\right)\sqrt{1-y^{2}}\frac{dy}{y-x}\\ \;\;\;\;\;\;\;\;\;\;+\frac{1}{\pi }\text{pv}\int_{b}^{c}g\left(y\right)\sqrt{1-y^{2}}\frac{dy}{y-x}\\ \;\;\;\;\;\;\;\;\;\;+\frac{1}{\pi }\text{pv}\int_{c}^{1}g\left(y\right)\sqrt{1-y^{2}}\frac{dy}{y-x}. \end{array}$$

Denote by *h*_1_ (*x*), *h*_2_ (*x*), *h*_3_ (*x*), and *h*_4_ (*x*) the four integrals on the right-hand side of (7), respectively. In other words,

(8) $$\sqrt{1-x^{2}}f\left(x\right)=C_{f}+h_{1}\left(x\right)+h_{2}\left(x\right)+h_{3}\left(x\right)+h_{4}\left(x\right)$$.

For *a* < *x* < *b*, the left-hand side of (8) is known. By our assumptions, *h*_2_ (*x*) and *h*_3_ (*x*) are known for any *x*. Therefore,

(9) $$h_{1}\left(x\right)+h_{4}\left(x\right)=\sqrt{1-x^{2}}f\left(x\right)-C_{f}-h_{2}\left(x\right)-h_{3}\left(x\right)$$

is known for *a* < *x* < *b*. Note that for *a* < *x* < *c*,

(10) $$\begin{array}{l} h_{1}\left(x\right)=\frac{1}{\pi }\int_{-1}^{a}g\left(y\right)\sqrt{1-y^{2}}\frac{dy}{y-x},\\ h_{4}\left(x\right)=\frac{1}{\pi }\int_{c}^{1}g\left(y\right)\sqrt{1-y^{2}}\frac{dy}{y-x} \end{array}$$

are given by ordinary integrals, because *y* − *x* ≠ 0 for −1 < *y* < *a* and *c* ≤ *y* < 1. Let us define complex functions *h*_1_ (*z*) and *h*_4_ (*z*) for *z* ∈ ℂ as

(11) $$\begin{array}{l} h_{1}\left(z\right)=\frac{1}{\pi }\int_{-1}^{a}g\left(y\right)\sqrt{1-y^{2}}\frac{dy}{y-z},\\ h_{4}\left(z\right)=\frac{1}{\pi }\int_{c}^{1}g\left(y\right)\sqrt{1-y^{2}}\frac{dy}{y-z}. \end{array}$$

By the Cauchy integral theorem, *h*_1_ (*z*), *h*_4_ (*z*), and hence *h*_1_ (*z*) + *h*_4_ (*z*) are analytic on the complex plane ℂ with cuts along the real axis from −∞ to *a* and from *c* to +∞. In particular, *h*_1_ (*z*) + *h*_4_ (*z*) is analytic on the real interval (*a*, *c*). From (9), *h*_1_ (*x*) + *h*_4_ (*x*) is known on (*a*, *b*), and the right-hand side of (9) is also analytic on (*a*, *b*). Note that *f*(*x*) is not an analytic function but *f*_1_(*x*) = $\sqrt{1-x^{2}}$ *f*(*x*) − *C*_*f*_ − *h*_2_ (*x*) − *h*_3_ (*x*) can be extended to an analytic function *f*_1_(*z*) in a neighborhood of (*a*,*b*). Since *h*_1_ (*z*) + *h*_4_ (*z*) is an analytic function on (*a*, *c*), the known analytic function *f*_1_ (*z*) can be analytically continued from (*a*, *b*) to (*a*, *c*). In other words, *h*_1_ (*x*) + *h*_4_ (*x*) is now known on (*a*, *c*). Using (8), *f*(*x*) can now be uniquely reconstructed since *h*_2_ (*x*) and *h*_3_ (*x*) are known on (*a*, *c*) as well. This proves Theorem 1.

Now let us study the stability of this reconstruction approach and estimate its error bound. Suppose that the function *f*(*x*) is measured as *f*_*ɛ*_ (*x*) with a measurement noise *ɛ*_*f*_ (*x*) by

(12) $$f_{\varepsilon }\left(x\right)=f\left(x\right)+\varepsilon_{f}\left(x\right)\;\;\;\;\text{for}\;a<x<b,$$

with

(13) $$\left|\varepsilon_{f}\left(x\right)\;\right|<\;\varepsilon \;\;\;\;\text{for}\;a<x<b\;,$$

where *ɛ* > 0 is a small number. We also assume that the backprojection (2) produces an error bounded by *ɛ*. In terms of the Hilbert transform,

(14) $$g_{\varepsilon }\left(x\right)=g\left(x\right)+\varepsilon_{g}\left(x\right)\;\;\;\;\text{for}\;-1<x<1,$$

with

(15) $$\left|\varepsilon_{g}\left(x\right)\;\right|<\;\varepsilon \;\;\;\;\text{for}\;-1<x<1.$$

We expect that the variation rate of the error term *ɛ*_*g*_ (*x*) is small. This can be seen from the fact that *g*(*x*) as a backprojection in (2) is defined by an integral and hence by an averaging process. This can also be seen from a data sampling point of view. The data sampling will lead to a small variation rate of *ɛ*_*g*_ (*x*) in a stochastic sense. Recall that

(16) $$h_{2}\left(x\right)+h_{3}\left(x\right)=\frac{1}{\pi }\text{pv}\int_{a}^{c}g\left(y\right)\sqrt{1-y^{2}}\frac{dy}{y-x}.$$

Rewriting the PV integral in (15), we have

(17) $$\begin{array}{l} h_{2}\left(x\right)+h_{3}\left(x\right)=\frac{1}{\pi }\text{pv}\int_{a}^{c}\frac{g\left(y\right)\sqrt{1-y^{2}}-g\left(x\right)\sqrt{1-x^{2}}}{y-x}dy\\ \;\;\;\;\;\;\;\;\;\;\;\;\;\;\;\;\;\;\;\;\;\;\;\;\;\;\;\;\;\;\;\;\;\;\;\;+\frac{g\left(x\right)\sqrt{1-x^{2}}}{\pi }pv\int_{a}^{c}\frac{dy}{y-x}\\ \;\;\;\;\;\;\;\;\;\;\;\;\;\;\;\;\;\;\;\;\;\;\;\;\;\;\;\;\;\;\;=\frac{1}{\pi }\text{pv}\int_{a}^{c}\frac{g\left(y\right)\sqrt{1-y^{2}}-g\left(x\right)\sqrt{1-x^{2}}}{y-x}dy\\ \;\;\;\;\;\;\;\;\;\;\;\;\;\;\;\;\;\;\;\;\;\;\;\;\;\;\;\;\;\;\;\;\;\;\;+\frac{g\left(x\right)\sqrt{1-x^{2}}}{\pi }\log\left(\frac{c-x}{x-a}\right). \end{array}$$

Let us change *g*(*x*) to *g*_*ɛ*_ (*x*) as in (14). Then *h*_2_ (*x*) + *h*_3_ (*x*) will become

(18) $$h_{23\varepsilon }\left(x\right)=h_{2}\left(x\right)+h_{3}\left(x\right)+\varepsilon_{h23}\left(x\right)\;\;\;\;\text{for}\;a<x<c$$,

where the error term is bounded by

(19) $$\left|\varepsilon_{23h}\left(x\right)\right|<C_{\varepsilon }+\frac{\varepsilon }{\pi }\left|\log\left(\frac{c-x}{x-a}\right)\right|,$$

where the relationship in (17) and the bound in (15) have been used. Here *C* is a constant. Note that this error bound becomes large when *x* is close to *c*. This suggests that one should only seek to reconstruct *f*(*x*) on [*b*, *c*_*δ*_] with *c*_*δ*_ < *c* appropriately. The right-hand side of (19) also becomes large when *x* is close to *a*. This will not cause any problem since *f*(*x*) is known on (*a*, *b*).

To determine the stability of the analytic continuation of *f*_1_ (*z*) = *h*_1_ (*z*) + *h*_4_ (*z*) from (*a*, *b*) to (*a*, *c*), we point out that, different from *f*_1_ (*x*), the measured function with error term *ɛ*_*f*1_ (*x*),

(20) $$f_{1\varepsilon }\left(x\right)=\sqrt{1-x^{2}}f_{\varepsilon }\left(x\right)-C_{f}-h_{23\varepsilon }\left(x\right)=f_{1}\left(x\right)+\varepsilon_{f1}\left(x\right),$$

with *f*_*ɛ*_ (*x*) and *h*_23*ɛ*_ (*x*) as in (12) and (18), cannot be extended to an analytic function. The stability of the analytic continuation of *f*_1_ (*z*) thus depends on the numerical method used. In Section 4, we will use the projection onto the convex sets (POCS) method [24] to compute the analytic continuation and *f*(*x*) from the measured data *f*_1*ɛ*_ (*x*). The stability of our algorithm therefore follows from that of POCS. In view of (20), (12), (13), (18), and (19), the reconstruction error is bounded by

(21) $$\sqrt{1-x^{2}}\left|f_{\varepsilon }\left(x\right)-f\left(x\right)\right|\leq C_{1}\varepsilon +C_{2}\varepsilon \left|\log\left(\frac{c-x}{x-a}\right)\right|.$$

The following comments are in order on the above theorem: first, no information on *f*(*x*) and *g*(*x*) is needed on [−1,*a*] and [*c*, 1], hence we are truly dealing with a truncated Hilbert transform. Second, the method employed in [9] can be adapted to reconstruct *f*(*x*) on [*b*, *c*) directly, and more sophisticated algorithms may be designed in the future. Third, although *μ* $\left(\vec{r}\right)$ is assumed to be a 2D function, Theorem 1 can be readily applied in the 3D case. Fourth, for practical implementation, both *f*(*x*) and *g*(*x*) are discretized at fine steps. Regarding the assumption of the finite-length interval (*a*, *b*), it can be as small as the sampling step so that *f*(*x*) can only be known on one sampling point inside the interval (*a*, *b*)!

From Theorem 1, we have the following corollaries.

**Corollary 1.**

*Let a*, *b, c, d be four real numbers with −1 < a < b< c < d <1. A function f(x) supported on [−1,1] can be exactly reconstructed on (a, b] and [c, d) if (i) f(x) is known on (b, c); (ii) g(x) is known on (a, d), and (iii) the constant C_f_ is known.*

**Corollary 2.**

*Let a* *, b, c, d be four real numbers with −1 < a < b < c < d < 1. A function f(x) supported on [−1,1] can be exactly reconstructed on [b, c] if (i) f(x) is known on (a, b) and (c, d), (ii) g(x) is known on (a, d), and (iii) the constant C_f_ is known.*

The proofs and stability analysis of Corollaries 1 and 2 can be made similar to that for Theorem 1. Under the same assumption, the reconstructed error of Corollary 2 is bounded by

(22) $$\sqrt{1-x^{2}}\left|f_{\varepsilon }\left(x\right)-f\left(x\right)\right|\leq C_{3}\varepsilon$$.

In fact, for *b* ≤ *x* ≤ *c* in Corollary 2, the corresponding term of |log((c−x)/(x−a))| in (21) is bounded. Note that there is no term which can go to infinity. This better control of reconstruction error is a main advantage of this reconstruction scheme with *f*(*x*) being known on two intervals.

## 3. DATA SUFFICIENT CONDITION AND RECONSTRUCTION APPROACH

From Theorem 1, we immediately have the following new data sufficient condition for exact and stable reconstruction of an ROI from limited projection data.

**Condition 1.**

The function *μ* can be exact reconstructed at a point $\vec{r}$_0_ if one can find a unit vector $\vec{n}$ = (−sin *Φ*_0_, cos *Φ*_0_) and a simply connected segmented *L*_*μ*_ ⊂ *L* of the line *L* parallel to $\vec{n}$ through $\vec{r}$_0_ such that (i) the segment *L*_*μ*_ contains $\vec{r}$_0_ and a segment *L*_0_ ⊂ *L*_*μ*_ on which the function *μ* is known, and (ii) for each $\vec{r}$ ∈ *L*_*μ*_ and each angle *Φ* ∈ [*Φ*_0_, *Φ*_0_ + *π*] the line integral *p*(*s*,*Φ*) are known for a neighborhood of *s* = $\vec{r}$ · $\vec{u}$(*Φ*).

**Figure 2:**
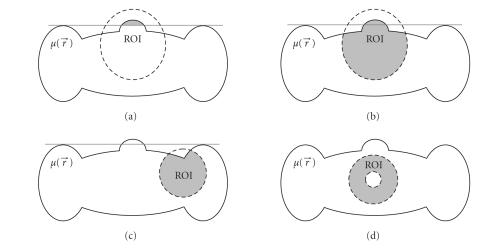
Circular field of view (FOV) and recoverable regions of interest (ROI) according to different data sufficiency conditions. (a) A small recoverable region per the condition by Noo et al., (b) the enlarged recoverable region per the condition by Defrise et al., (c) and (d) the recoverable region per our new data sufficiency condition when the FOV is contained in the convex hull of the compact support of the object and covers a part of known region. The dashed lines outline the FOV. The gray-region represents the recoverable ROI, where the exact reconstruction can be performed.

To illustrate our above condition, let us define the field of view (FOV) Λ ⊂ ℝ^2^ as follows: for any $\vec{r}$ ∈ Λ and *Φ* ∈ [0,*π*), there exists an *s* satisfying *p*(*s*, *Φ*) through the point $\vec{r}$. It is well known that a necessary condition for exact reconstruction of an ROI is that the ROI must be contained in the FOV of a CT scan. Now, we consider circular FOVs as shown in Figure 2. Traditionally, to reconstruct an ROI exactly, all the lines going through the compact support of the object function should be measured [25], which indicates that the recoverable region is empty for all the cases. The condition by Noo et al. [7] allows that *μ* $\left(\vec{r}\right)$ at any point $\vec{r}$ inside the FOV is recoverable if there exists a line through $\vec{r}$ and the intersection between the line and compact support of *μ* $\left(\vec{r}\right)$ is completely contained in the FOV. Hence, we can have a small recoverable ROI as shown in Figure 2(a). The condition by Defrise et al. [9] claims that *μ* $\left(\vec{r}\right)$ at any point $\vec{r}$ inside the FOV is recoverable if there exists a line segment in the FOV through $\vec{r}$ and at least one of its ends is outside the convex hull of the object support. In contrast to the condition by Noo et al., the recoverable ROI is greatly enlarged as in Figure 2(b). Our new data sufficient condition states that *μ* $\left(\vec{r}\right)$ at any point $\vec{r}$ inside the FOV is recoverable if there exists a line segment in the FOV through $\vec{r}$ and the function *μ* is known on part of that line segment. Clearly, the condition of Defrise et al. is a special case of ours. Defreise et al. require that the known part, which equals to zero, should be outside of the convex hull of the compact support. In fact, this is unnecessary according to our new data sufficiency condition. The known part can be inside the convex hull with *μ* $\left(\vec{r}\right)$ = 0 or even inside the compact support with *μ* $\left(\vec{r}\right)$ is known, as shown in Figures 2(c) and 2(d), respectively. It should be pointed out that the above analysis can be directly extended to the 3D case for VOI.

To reconstruct the object function inside an FOV satisfying our data sufficient condition, a general reconstruction approach is given in the following steps:

- construct a group of line segments each of which goes through both known and unknown regions;
- reconstruct the unknown region based on Theorem 1 in Section 2;
- repeat Steps (i) and (ii) until the object function at all eligible points inside the FOV is reconstructed.

**Figure 3:**
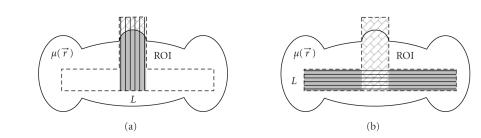
Illustration of the general exact ROI reconstruction approach in the case of a T-shaped ROI. (a) Per the data sufficiency condition by Defrise et al., a group of vertical line segments can be exactly and stably reconstructed; and (b) per our new data sufficiency condition, a group of horizontal line segments can also be exactly and stably reconstructed using our truncated Hilbert transform technology. The textured regions are known before the involved truncated Hilbert transforms are performed.

Our approach works like a water stream flowing from a known region to all the connected unknown zones subject to our data sufficiency condition. Figure 3 illustrated this procedure with a T-shaped ROI. Note that using our approach there are multiple ways to perform exact ROI/VOI reconstructions, suggesting opportunities for further theoretical and numerical studies.

## 4. SIMULATION RESULTS

Similar to what Defrise et al. did in [9], we computed the inversion of the truncated Hilbert transform as used in Theorem 1 using the projection onto convex sets (POCS) method [24]. Using the notation in Section 2, our goal is to determine a second-order continuous function *f*(*x*)∈ *L* ^2^(ℝ) in the intersection of the following five convex sets:

- *C*_1_ = {*f* ∈ *L* ^2^(ℝ) ∣ (*Hf*)(*x*) = *g*(*x*), *x* ∈ (*a*, *c*) },
- *C*_2_ = {*f* ∈ *L* ^2^(ℝ) ∣ *f*(*x*) = *f*_0_ (*x*), *x* ∈ (*a*, *b*)},
- *C*_3_ = {*f* ∈ *L* ^2^(ℝ) ∣ (1/*π*) ∫^1^_−1_ *f*(*x*)*dx* = *C*_*f*_},
- *C*_4_ = {*f* ∈ *L* ^2^(ℝ) ∣ *f*(*x*) ≥ 0, *x* ∈ [−1,1] },
- *C*_5_ = {*f* ∈ *L* ^2^(ℝ) ∣ *f*(*x*) ≥ *f*_max_, *x* ∈ [−1,1] },

where *f*_0_ (*x*) is the known part, and *f*_max_ is the upper bound of *f*(*x*). With an initial guess of the unknown function, which can be constructed over the known object support, the POCS algorithm iteratively projects an intermediate solution to each of the above five convex sets until it converges to a satisfactory result.

**Figure 4:**
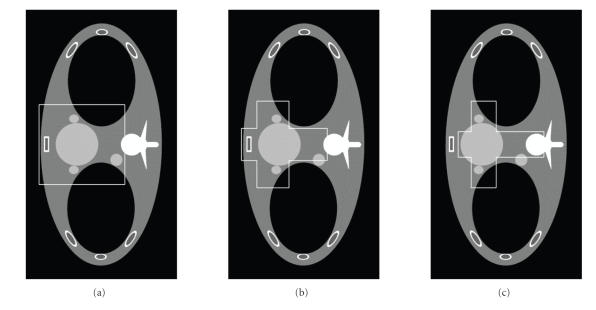
Representative slice of the thorax phantom reconstructed from a complete dataset. (a) A rectangular ROI for reproducing the results by Defrise et al.; (b) and (c) are two cross-shaped ROIs for evaluating our truncated Hilbert transform technology.

**Figure 5:**
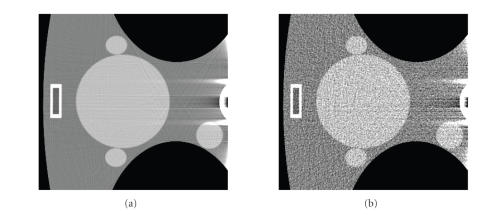
Reconstructed results in the rectangular ROI indicated in Figure 4(a) using the approach developed by Defrise et al. (a) Reconstructed ROI from noise-free data, and (b) the counterpart from noisy data. The display window is [0.9,1.1].

**Figure 6:**
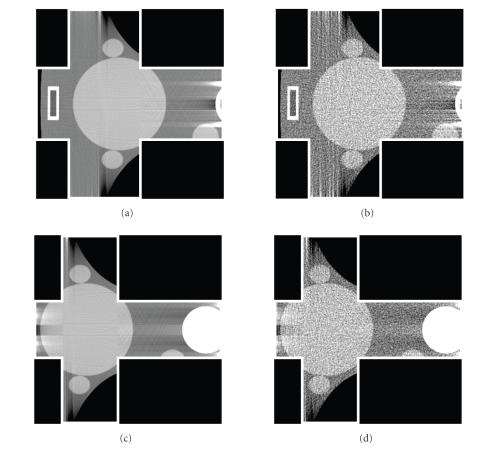
Reconstructed results in the cross-shaped ROIs indicated in Figures 4(b) and 4(c) using our approach. (a) and (c) are reconstructed from noise-free data. (b) and (d) are the counterpart from noisy data. The display window is [0.9,1.1].

The above POCS method was numerically implemented in Matlab to demonstrate the correctness of our data sufficient condition and generalized reconstruction framework. As illustrated in Figure 4, the function *μ* $\left(\vec{r}\right)$ is an axial slice of the FORBILD thorax phantom [26] with two small ellipses added to the heart to make it more challenging for reconstruction, which was also used in the paper by Defrise et al. [9]. Nontruncated fan-beam projection data of 1200 directions were analytically computed over a full-scan range. Hence, the backprojection function *g* at any point can be calculated along any line to simulate different FOV configurations. First, we repeated the work by Defrise et al. [9] to reconstruct a rectangular ROI indicated in Figure 4(a) from noise-free projection data. Then, we reconstructed two cross-shaped ROIs in Figures 4(b) and 4(c) using our approach proposed in Section 3. While in Figure 4(b) we used the prior information that the reconstructed function was zeros outside its compact support, we assumed that the central part of the cross-shaped ROI was known in Figure 4(c). To test the stability of our method, the above results were repeated from noisy data with 2 × 10^5^ photons per incident ray. The representative images were presented in Figures 5 and 6. As compared to the results in [9], our reproduced image quality in Figure 5 seemed better. The possible reasons include (a) the condition *f*(*x*) ≥ *f*_max_ was used for the POCS method with *f*_max_ = 2, and (b) 400 iterations was executed, which is twice that in [9]. As seen in Figures 5 and 6, the reconstructed image quality in the cross-shaped ROIs is very comparable to that in the rectangular ROI. This validated our data sufficient condition and general ROI reconstruction approach.

## 5. DISCUSSIONS AND CONCLUSIONS

While our work has been presented in the context of X-ray CT and micro-CT, we underline that the significance and implication of our results are far beyond what has been described above. The same or similar techniques can be applied for X-ray phase-contrast imaging and tomography, emission tomography including PET and SPECT, and other modalities that rely on a projective imaging model. Our proposed approach can be used not only for exact reconstruction of an ROI/VOI but also for approximate reconstruction of various types. Furthermore, new lambda tomography techniques may be developed based on the truncated Hilbert transform theory proposed in this paper and will be further refined in the future. The conventional wisdom has been that the exact and stable reconstruction of an ROI/VOI inside an object support is generally impossible from truly truncated data that go only through the ROI/VOI. However, according to our new data sufficiency condition, such an exact and stable reconstruction becomes feasible if a small subregion is known inside the ROI/VOI, even though the projection data remain truly truncated!

In conclusion, we have presented a general ROI/VOI reconstruction approach using a truly truncated Hilbert transform on a segment of a chord inside a compactly supported object aided by partial knowledge on one or both neighboring intervals of that segment. Our approach and associated new data sufficient condition allows the most flexible ROI/VOI image reconstruction from the minimum account of data in both the fan-beam and cone-beam geometry. We are actively working along this direction to realize major theoretical potentials and enable innovative practical applications.
